# Prognostic nomogram for cancer-specific survival in adenosquamous carcinoma of lung patients treated with chemotherapy: A SEER-based retrospective cohort study

**DOI:** 10.1097/MD.0000000000047538

**Published:** 2026-02-13

**Authors:** Jinyuan Xiao, Jiarun Fan, Maoping Wang, Xiaoliang Yuan

**Affiliations:** a Gannan Medical University, Ganzhou, Jiangxi, China; b Department of Respiratory Medicine, The First Affiliated Hospital of Gannan Medical University, Ganzhou, Jiangxi, China.

**Keywords:** adenosquamous carcinoma of lung, chemotherapy, nomogram, SEER database

## Abstract

This study aims to establish a prognostic nomogram among adenosquamous carcinoma of lung (ASC) patients who received chemotherapy. A total of 704 ASC patients who received chemotherapy were extracted from the Surveillance, Epidemiology, and End Results database. Patients were randomly divided into a training (n = 493) and a validation (n = 211) cohort. Cancer-specific survival (CSS) was the primary endpoint of this study. First, independent prognostic predictors of CSS identified from univariate and multivariate Cox regression analyses were used to construct a prognostic nomogram for predicting 1-, 3-, and 5-year CSS in those patients. Then, calibration curves and receiver operating characteristic curves were used to evaluate the nomogram’s prediction accuracy, while decision curve analysis was used to evaluate the nomogram’s clinical utility. Finally, a mortality risk stratification system was constructed for this subpopulation. Eight clinical parameters were identified as independent prognostic factors for ASC patients who received chemotherapy, including age, household income, T stage, N stage, bone metastasis, brain metastasis, surgery, and the primary site of the tumor. The calibration curves, receiver operating characteristic, and decision curve analysis showed that the nomogram had excellent discrimination and clinical value. Moreover, the mortality risk stratification system could effectively divide all patients into 3 risk subgroups and achieve targeted patient management. Based on the Surveillance, Epidemiology, and End Results database, a novel prognostic nomogram for predicting 1-, 3-, and 5-year CSS in patients with ASC that underwent chemotherapy has been constructed and validated. The nomogram showed relatively good performance, which could be used in clinical practice to assist clinicians in individualized treatment strategies.

## 1. Introduction

Lung cancer is currently the most common and deadly malignant tumor worldwide. According to the most recent statistical data, approximately 2.5 million new cases of lung cancer were reported, accounting for 12.4% of all malignant tumors. The number of new deaths was approximately 1.8 million, representing 18.7% of the total.^[1]^ Lung cancer can be histologically classified into 2 major categories: small cell lung cancer and non-small cell lung cancer (NSCLC). NSCLC can be further subclassified into adenocarcinoma (ADC), squamous cell carcinoma (SCC), adenosquamous carcinoma (ASC), and other rarer forms of lung cancer.^[2]^

ASC is a relatively rare histological subtype of NSCLC, accounting for only 0.4% to 4.0% of all lung cancer cases.^[3]^ In 1999, the WHO standardized the definition of ASC. ASC is a type of cancer that comprises both SCC and ADC components. When observed under a light microscope, each component accounts for at least 10% of the tumor.^[4]^ Compared with ADC and SCC, ASC demonstrates greater aggressiveness and a poorer prognosis.^[5]^ Despite the breakthroughs achieved by targeted therapy and immunotherapy in the field of lung cancer in recent years, chemotherapy, as a traditional treatment modality, still holds an irreplaceable position in the comprehensive management of ASC.^[6]^ As a fundamental therapeutic approach for malignant tumors, chemotherapy exhibits a certain degree of efficacy in ASC patients. However, its inability to achieve a complete cure of cancer and the associated significant toxic reactions render the application of chemotherapy a complex issue.^[7]^ Therefore, identifying key factors influencing chemotherapy efficacy and integrating them to optimize patient management strategies is crucial for enhancing treatment effectiveness, improving quality of life, and advancing personalized medicine. This study, based on Surveillance, Epidemiology, and End Results (SEER) data, aims to identify critical prognostic factors for pulmonary ASC patients undergoing chemotherapy and develop a nomogram for visual prognosis prediction.

## 2. Methods

### 2.1. Database

The data used in this study were all sourced from the SEER database and downloaded using the SEER*Stat software (version 8.4.2), which is freely available on the SEER database website (https://seer.cancer.gov). The SEER database is a publicly accessible database collected and maintained by the National Cancer Institute of the United States since 1973. It covers approximately 48% of cancer patients in the United States and records their diagnosis, treatment, and survival data. The information collected regularly includes patients’ demographic characteristics, primary tumor information, treatment plans, as well as clinical retrospective data on survival status and survival time. The SEER database is a public data resource, and since this study did not involve patients’ specific identity information but only extracted their basic clinical information, it did not require approval from an ethics review committee.

### 2.2. Population selection

**Figure 1.**
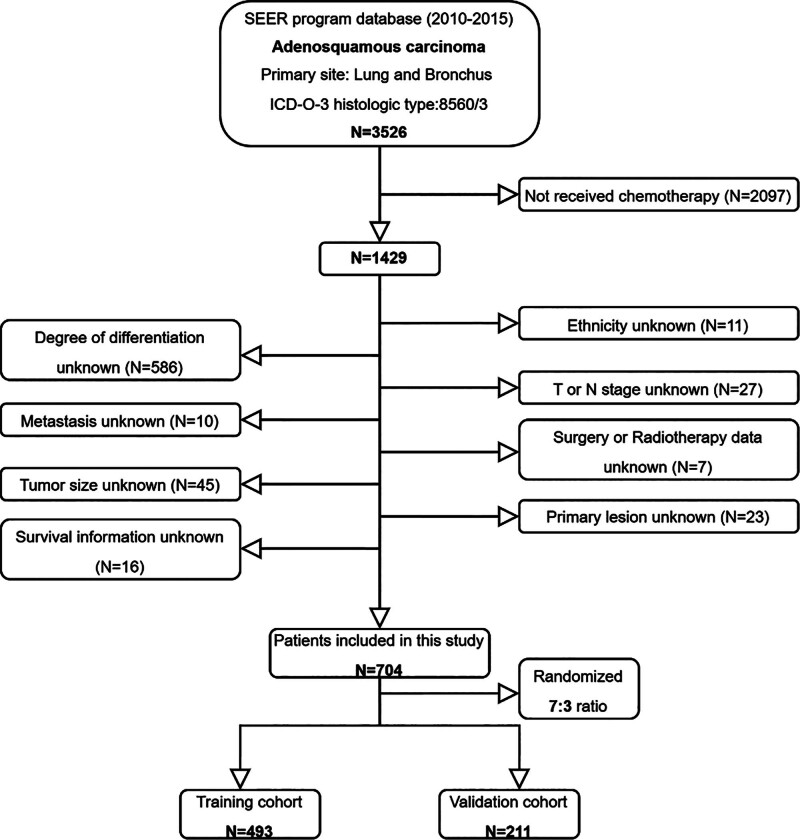
Flowchart for the screening of patients with ASC included. ASC = adenosquamous carcinoma of lung, SEER = Surveillance, Epidemiology, and End Results.

The inclusion criteria included: Patients with histologically confirmed ASC during the period from 2010 to 2015; ICD-O-3 histology code: 8560/3; primary tumor; and with clear follow-up data. The exclusion criteria were as follows: Not received chemotherapy and incomplete clinical information. Ultimately, this study identified 704 eligible patients with ASC who underwent chemotherapy from the SEER database. They were randomly allocated into a training cohort and a validation cohort in a 7:3 ratio (Fig. 1).

### 2.3. Study variables

Patients were stratified based on multiple demographic, clinicopathological, and treatment-related factors. Demographic variables included age (≤65 and > 65 years old), sex (male and female), race (White, Black, or other), marital status (married; divorced/widowed/separated; or unmarried/single/unknown), household income (<50,000; 50,000–64,999; and ≥ 65,000), and residential area (metropolitan [>1 million population], urban [<1 million], and rural/nonurban). Clinicopathological characteristics encompassed diagnosis year (2010–2012 and 2013–2015), tumor size (≤30, 31–50, 51–70, and > 70 mm), differentiation grade (I, well-differentiated; II, moderately differentiated; III, poorly differentiated; and IV undifferentiated), T stage (T1–T4), N stage (N0–N3), anatomical location (upper lobe, middle lobe, lower lobe, main bronchus, and cross-lobar), and lateral distribution (left, right, and bilateral). Metastasis status was categorized for bone, brain, liver, and lung involvement (presence and absence). Treatment modalities included radiotherapy (yes and no) and surgery (yes and no). The primary endpoint was cancer-specific survival (CSS), defined as the interval from diagnosis to tumor-related death.

### 2.4. Statistical analysis

 All data were analyzed using SPSS (version 25.0; SPSS, an IBM Company, Chicago) and R (version 4.3.2; The R Foundation for Statistical Computing, Vienna, Austria) software. A *P*-value of <.05 was considered statistically significant. CSS served as the primary endpoint of this study, defined as the time interval between the date of diagnosis and the time of death solely attributed to the tumor. Hazard ratios (HR) and their corresponding 95% confidence intervals (CI) were employed to demonstrate the impact of the selected variables on CSS in these patients. Initially, a univariate Cox model analysis was conducted to identify statistical differences among the variables. Subsequently, variables with statistical significance were chosen for multivariate Cox regression analysis to eliminate confounding effects and identify independent predictors associated with CSS. Based on these predictors, a nomogram was constructed to predict the 1-, 3-, and 5-year survival rates of patients with ASC undergoing chemotherapy. To evaluate the performance of the constructed nomogram and to test for overfitting, internal validation was performed using the bootstrap resampling method with 1000 repetitions. A bootstrap-corrected C-index was calculated. Additionally, calibration curves for 1-, 3-, and 5-year CSS were plotted to visually assess the agreement between predicted probabilities and actual observations. In addition, the time-dependent area under the curve (AUC) values of the 1-, 3-, and 5-year receiver operating characteristic (ROC) curves were used to evaluate the discriminatory ability of the nomogram. Furthermore, ROC curves for all independent predictors were plotted to verify that the predictive validity of the constructed nomogram was superior to that of individual independent predictors. The decision curve analysis (DCA) were utilized to validate the clinical benefit of the nomogram. The risk score was calculated using CSS-related independent prognostic factors, with optimal cutoffs determined via X-tile software. Patients were stratified into low-, intermediate-, and high-risk groups, and CSS differences among them were compared using the Kaplan–Meier method.

## 3. Results

### 3.1. Demographic and clinicopathologic characteristics

Based on the inclusion criteria, a total of 3526 patients with ASC were identified from the SEER database, among whom 1429 (40.5%) ASC patients received chemotherapy. According to the exclusion criteria, 725 patients were excluded. Subsequently, 704 patients were further allocated to the training and validation cohorts in a 7:3 ratio. The baseline characteristics of the cohort (n = 704) were as follows: 61.5% aged ≥ 65 years; 36.4% had tumor diameters of 31 to 50 mm; males accounted for 52.6%. Ethnicity was predominantly White (80.8%), with 58.0% married and 43.6% reporting annual household income ≥$65,000. Poorly differentiated tumors were common (72.6%). T2 (39.9%) and N2 (42.0%) stages were most frequent; distant metastases occurred in bone (12.5%), brain (9.8%), liver (5.4%), and lung (10.9%). Geographically, 57.0% resided in urban areas (>1 million population). Primary tumor sites were mainly upper lobe (57.5%) and lower lobe (34.7%), with 99.6% unilateral. No significant differences in surgery or radiotherapy rates were observed. Baseline characteristics were well-balanced between training and validation groups (Table 1).

**Table 1:** Demographic, clinicopathological characteristics, and treatment information for the training and validation groups.

Variables	Training cohort	Validation cohort	Total	*P* value
N	493	211	704	
Age (yr)				.293
<65	196 (39.8%)	75 (35.5%)	271 (38.5%)	
≥65	297 (60.2%)	136 (64.5%)	433 (61.5%)	
Tumor size (mm)				.906
≤30	141 (28.6%)	55 (26.1%)	196 (27.8%)	
31–50	178 (36.1%)	78 (37.0%)	256 (36.4%)	
51–70	95 (19.3%)	44 (20.9%)	139 (19.7%)	
>70	79 (16.0%)	34 (16.1%)	113 (16.1%)	
Sex (n,%)				.411
Male	254 (51.5%)	116 (55.0%)	370 (52.6%)	
Female	239 (48.5%)	95 (45.0%)	334 (47.4%)	
Race (n,%)				.964
White	398 (80.7%)	171 (81.0%)	569 (80.8%)	
Black	45 (9.1%)	18 (8.5%)	63 (8.9%)	
Other	50 (10.1%)	22 (10.4%)	72 (10.2%)	
Marital status (n,%)				.999
Married	286 (58.0%)	122 (57.8%)	408 (58.0%)	
Divorced	123 (24.9%)	53 (25.1%)	176 (25.0%)	
Single	84 (17.0%)	36 (17.1%)	120 (17.0%)	
Household income (n,%)				.797
<$50,000	101 (20.5%)	42 (19.9%)	143 (20.3%)	
$50,000–$64,999	181 (36.7%)	73 (34.6%)	254 (36.1%)	
≥$ 65,000	211 (42.8%)	96 (45.5%)	307 (43.6%)	
Year of diagnosis (n,%)				.461
2010–2012	251 (50.9%)	109 (51.7%)	360 (51.1%)	
2013–2015	242 (49.1%)	102 (48.3%)	344 (48.9%)	
Grade (n,%)				.313
Grade I	6 (1.2%)	4 (1.9%)	10 (1.4%)	
Grade II	121 (24.5%)	48 (22.7%)	169 (24.0%)	
Grade III	359 (72.8%)	152 (72.0%)	511 (72.6%)	
Grade IV	7 (1.4%)	7 (3.3%)	14 (2.0%)	
T stage (n,%)				.122
T1	78 (15.8%)	27 (12.8%)	105 (14.9%)	
T2	185 (37.5%)	96 (45.5%)	281 (39.9%)	
T3	147 (29.8%)	49 (23.2%)	196 (27.8%)	
T4	83 (16.8%)	39 (18.5%)	122 (17.3%)	
N stage (n,%)				.092d
N0	147 (29.8%)	44 (20.9%)	191 (27.1%)	
N1	89 (18.1%)	40 (19.0%)	129 (18.3%)	
N2	199 (40.4%)	97 (46.0%)	296 (42.0%)	
N3	58 (11.8%)	30 (14.2%)	88 (12.5%)	
Bone metastasis (n,%)				.456
No	428 (86.8%)	188 (89.1%)	616 (87.5%)	
Yes	65 (13.2%)	23 (10.9%)	88 (12.5%)	
Brain metastasis (n,%)				.268
No	449 (91.1%)	186 (88.2%)	635 (90.2%)	
Yes	44 (8.9%)	25 (11.8%)	69 (9.8%)	
Liver metastasis (n,%)				.384
No	464 (94.1%)	202 (95.7%)	666 (94.6%)	
Yes	29 (5.9%)	9 (4.3%)	38 (5.4%)	
Lung metastasis (n,%)				.984
No	439 (89.0%)	188 (89.1%)	627 (89.1%)	
Yes	54 (11.0%)	23 (10.9%)	77 (10.9%)	
residential area (n,%)				.474
Metropolitan	274 (55.6%)	127 (60.2%)	401 (57.0%)	
Urban	153 (31.0%)	61 (28.9%)	214 (30.4%)	
Rural/nonurban	66 (13.4%)	23 (10.9%)	89 (12.6%)	
Primary site (n,%)				.163
Upper lobe	280 (56.8%)	125 (59.2%)	405 (57.5%)	
Middle lobe	23 (4.7%)	4 (1.9%)	27 (3.8%)	
Lower lobe	174 (35.3%)	70 (33.2%)	244 (34.7%)	
Main bronchus	8 (1.6%)	8 (3.8%)	16 (2.3%)	
Overlapping lesion of lung	8 (1.6%)	4 (1.9%)	12 (1.7%)d	
Laterality (n,%)				.989
Left	216 (43.8%)d	93 (44.1%)	309 (43.9%)	
Right	275 (55.8%)	117 (55.5%)	392 (55.7%)	
Bilateral	2 (0.4%)	1 (0.5%)	3 (0.4%)	
Radiotherapy (n,%)				.307
No	245 (49.7%)	96 (45.5%)	341 (48.4%)	
Yes	248 (50.3%)	115 (54.5%)	363 (51.6%)	
Surgery (n,%)				.277
No	242 (49.1%)	113 (53.6%)	355 (50.4%)	
Yes	251 (50.9%)	98 (46.4%)	349 (49.6%)	

### 3.2. Independent predictors influencing the CSS in patients with ASC undergoing chemotherapy

In the training cohort (n = 493), a total of 325 CSS events were observed, which were used for the construction of the Cox proportional hazards model. The results of the univariate Cox regression analysis revealed statistically significant differences in CSS among patients for the following variables: age, household income, T stage, N stage, bone metastasis, brain metastasis, liver metastasis, lung metastasis, receipt of radiotherapy, receipt of surgery, and primary tumor site (Table 2). Compared to patients with grade I differentiation, those with grade IV differentiation had an HR of 1.773 (95% CI: 0.476–6.608, *P* = .394), which was not statistically significant. However, as reported in the literature,^[8–10]^ tumor differentiation grade is associated with the prognosis of ASC patients. Therefore, this factor was included in the further multivariate Cox regression analysis.

**Table 2:** Univariate and multivariate COX regression analysis of CSS in the training group.

Variables	Univariate analysis		Multivariate analysis	
	HR (95% CI)	*P* value	HR (95% CI)	*P* value
Age (yr)				
<65	Ref			
≥65	1.561 (1.240–1.965)	<.001*	1.424 (1.123–1.805)	.004*
Sex				
Male	Ref			
Female	0.827 (0.665–1.028)	.087		
Race				
White	Ref			
Black	1.367 (0.960–1.947)	.083		
Other	0.821 (0.559–1.205)	.314		
Marital status				
Married	Ref			
Divorced	1.061 (0.820–1.373)	.653		
Single	1.083 (0.802–1.464)	.601		
Household income				
<$50,000	Ref			
$50,000–$64,999	1.080 (0.810–1.440)	.600	0.813 (0.601–1.099)	.178
≥$65,000	0.738 (0.552–0.988)	.041*	0.576 (0.425–0.782)	<.001*
Year of diagnosis				
2010–2012	Ref			
2013–2015	1.111 (0.892–1.385)	.347		
Grade (n,%)				
Grade I	Ref			
Grade II	0.786 (0.287–2.157)	.640	1.021 (0.365–2.859)	.968
Grade III	1.340 (0.499–3.600)	.562	1.298 (0.474–3.555)	.611
Grade IV	1.773 (0.476–6.608)	.394	1.952 (0.512–7.438)	.327
T stage (n,%)				
T1	Ref			
T2	1.025 (0.725–1.448)	.889	1.253 (0.879–1.786)	.212
T3	1.379 (0.970–1.962)	.074	1.254 (0.871–1.808)	.224
T4	2.243 (1.534–3.279)	<.001*	1.628 (1.088–2.435)	.018*
N stage (n,%)				
N0	Ref			
N1	1.213 (1.012–1.534)	.014*	1.219 (0.801–1.620)	.213
N2	1.620 (1.247–2.103)	<.001*	1.590 (0.935–2.017)	.173
N3	2.259 (1.939–3.926)	<.001*	2.012 (1.569–3.012)	<.001*
Bone metastasis				
No	Ref			
Yes	4.205 (3.126–5.655)	<.001*	2.352 (1.687–3.279)	<.001*
Brain metastasis				
No	Ref			
Yes	2.403 (1.709–3.379)	<.001*	1.547 (1.063–2.253)	.023*
Liver metastasis				
No	Ref			
Yes	2.935 (1.955–4.408)	<.001*	0.996 (0.626–1.583)	.985
Lung metastasis				
No	Ref			
Yes	2.122 (1.545–2.915)	<.001*	0.941 (0.659–1.344)	.738
Radiotherapy				
No	Ref			
Yes	1.737 (1.393–2.167)	<.001*	1.128 (0.865–1.470)	.373
Surgery				
No	Ref			
Yes	0.263 (0.208–0.333)	<.001*	0.414 (0.33–-0.609)	<.001*
Residential area				
A city of over 1 million inhabitants	Ref			
A city of fewer than 1 million inhabitants	1.148 (0.902–1.462)	.262		
Counties, shires, or other nonurban administrative regions	1.078 (0.780–1.489)	.650		
Primary site				
Upper lobe	Ref			
Middle lobe	0.845 (0.481–1.485)	.559	0.936 (0.522–1.677)	.824
Lower lobe	1.354 (1.076–1.703)	.010*	1.332 (1.041–1.704)	.022*
Main bronchus	3.249 (1.594–6.621)	.001*	1.889 (0.909–3.924)	.088
Overlapping lesion of lung	0.808 (0.300–2.179)	.674	1.079 (0.396–2.944)	.882
Laterality				
Left	Ref			
Right	0.897 (0.721–1.117)	.331		
Bilateral	2.946 (0.726–11.952)	.130		

CI = confidence intervals, CSS = cancer-specific survival, HR = hazard ratios.

* *P* < .05 is considered statistically significant.

The statistically significant factors from the univariate Cox regression analysis, along with tumor differentiation grade, were further incorporated into the multivariate Cox regression analysis. The result showed that age, household income, T stage, N stage, bone metastasis, brain metastasis, surgery, and primary tumor site are independent predictive factors influencing the CSS of patients with ASC who receive chemotherapy (Table 2).

### 3.3. Construction and validation of a prognostic nomogram for patients with ASC receiving chemotherapy

**Figure 2.**
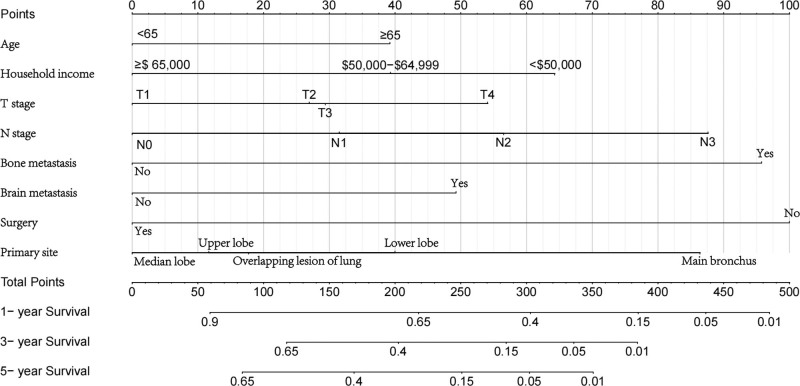
Nomogram for predicting 1-, 3-, and 5-year survival rates in ASC patients receiving chemotherapy. ASC = adenosquamous carcinoma of lung.

**Figure 3.**
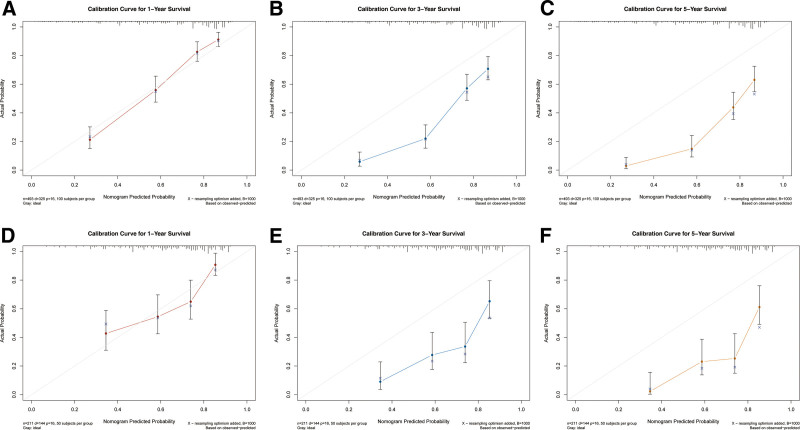
Calibration curves of the nomogram: 1 (A), 3 (B), and 5 (C) years in the training group and 1 (D), 3 (E), and 5 (F) years in the validation group.

**Figure 4.**
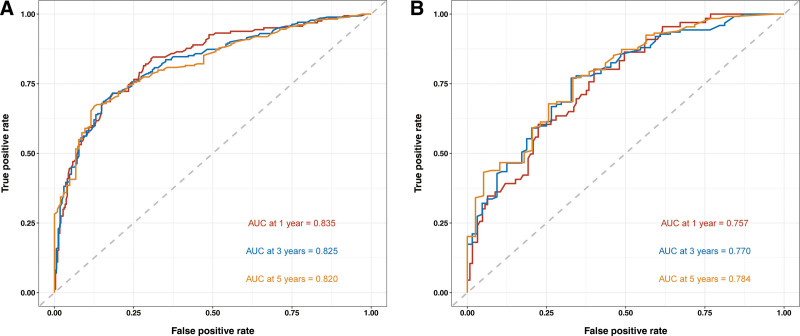
Receiver operating characteristic curves at 1, 3, and 5 years for ASC chemotherapy patients in the training (A) and validation (B) groups. ASC = adenosquamous carcinoma of lung, AUC = area under the curve.

**Figure 5.**
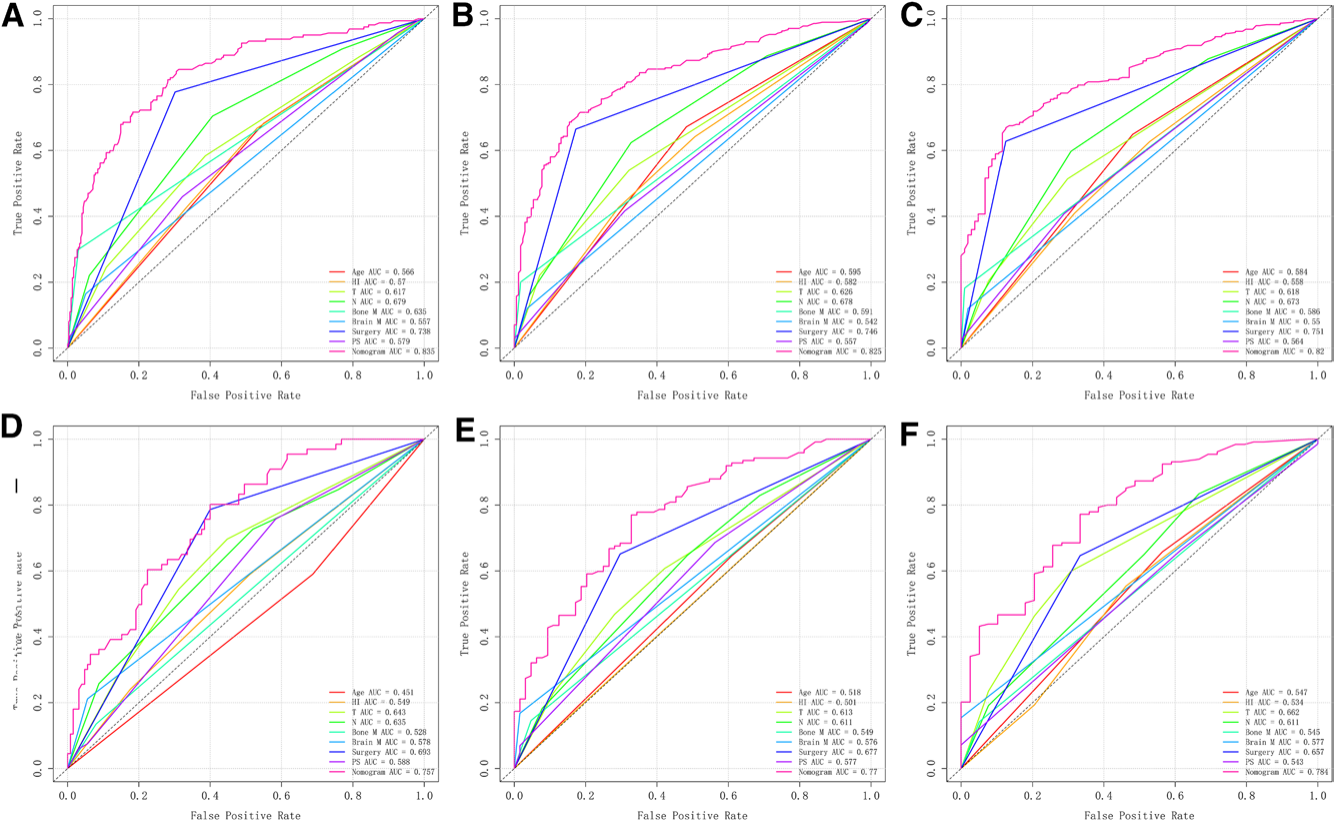
The predictive accuracy of the constructed novel nomogram was compared with the independent prognostic predictors associated with each CSS in ASC patients treated with chemotherapy at 1 (A), 3 (B), and 5 (C) years in the training group and 1 (D), 3 (E), and 5 (F) years in the validation group. ASC = adenosquamous carcinoma of lung, AUC = area under the curve, HI = household income, M = metastasis, PS = primary site.

**Figure 6.**
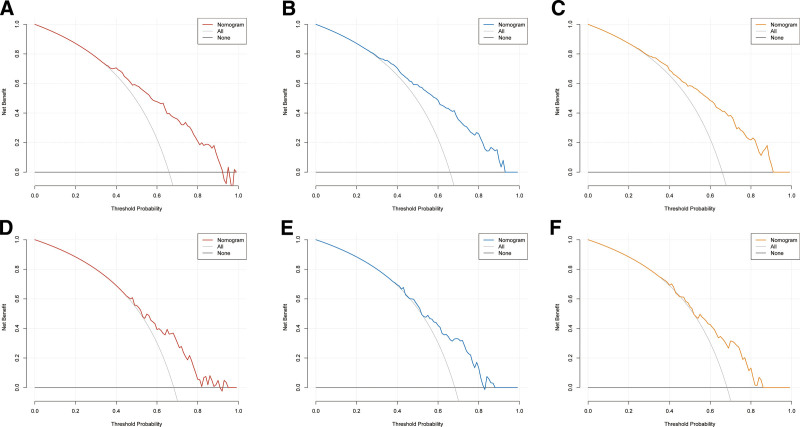
Decision curves of the nomogram: 1 (A), 3 (B), and 5 (C) years in the training group and 1 (D), 3 (E), and 5 (F) years in the validation group.

Based on multivariate Cox regression, 8 independent prognostic factors were identified to develop a predictive nomogram in chemotherapy-treated ASC patients (Fig. 2). From this nomogram, we can visually discern the degree of influence each variable has on the prognosis of ASC patients receiving chemotherapy.The model assigns corresponding scores to each independent prognostic determinant based on the weight coefficients determined by multivariate Cox regression analysis, with the total score obtained by summing the scores of each variable. By vertically mapping the total score axis to the survival probability axis of the nomogram, clinicians can quantitatively assess a patient’s 1-, 3-, and 5-year survival probabilities, enabling precise prognostic stratification. After internal validation using the Bootstrap resampling method (1000 iterations), the nomogram’s C-index was 0.747 (95% CI: 0.700–0.778), indicating good predictive performance. From the nomogram, it can be observed that surgery has the greatest impact on prognosis, followed by bone metastasis, N stage, and primary tumor site. The 1-, 3-, and 5-year calibration curves demonstrated good agreement between the actual and predicted outcomes (Fig. 3). The AUC for CSS in the training group was 0.835, 0.825, and 0.820 for 1-, 3-, and 5-year survival, respectively, while in the validation group, the AUCs were 0.757, 0.770, and 0.784, confirming the model’s robust discriminatory ability (Fig. 4). To validate the nomogram’s superior predictive performance, we compared its accuracy with individual CSS-related prognostic factors (Fig. 5). In both groups, the 1-, 3-, and 5-year AUCs of all independent factors were lower than those of the nomogram, underscoring the advantage of multivariate integration. DCA curves in both training and validation groups showed excellent positive net benefits across broad risk thresholds, indicating high clinical application value of the nomogram (Fig. 6).

### 3.4. Risk stratification system for patients with ASC receiving chemotherapy

**Figure 7.**
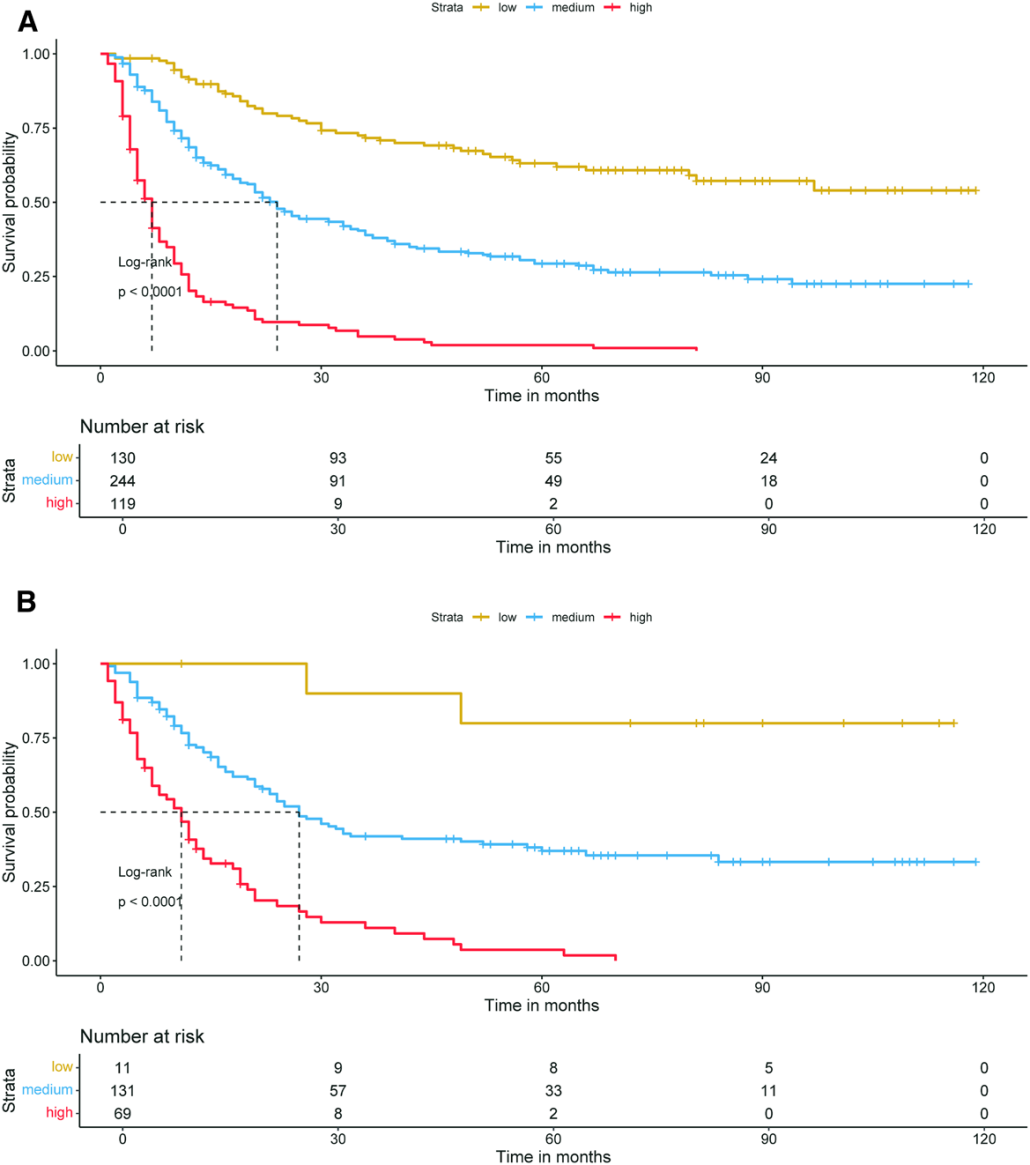
Kaplan–Meier survival analysis and the log-rank test were used to compare CSS in ASC chemotherapy patients in the 3 risk subgroups of the training group (A) and validation group (B). The high-risk subgroup had a worse prognosis than the low-risk subgroup. ASC = adenosquamous carcinoma of lung, CSS = cancer-specific survival.

To further validate the nomogram’s stability and performance, a cancer-specific mortality risk stratification system was constructed based on the 8 CSS-related independent prognostic predictors. Specifically, each patient’s total score was calculated. Using the X-tile algorithm, optimal total score cutoffs (49 and 248) were identified, classifying patients into low-risk (<49), intermediate-risk (49–248), and high-risk (>248) subgroups. Kaplan–Meier curves (Fig. 7) confirmed significant prognostic differences across subgroups in both groups. Patients with a high risk of mortality exhibited a poorer prognosis compared to those with a low risk of mortality, indicating that the CSS risk stratification system constructed using the prognostic nomogram possesses excellent predictive capability and facilitates improved management of cancer patients.

## 4. Discussion

Lung cancer is the most common malignant tumor globally, with NSCLC comprising approximately 85% of cases. Among NSCLC subtypes, ASC is a rare but aggressive mixed-cell neoplasm, combining clinical and biological features of both ADC and SCC.^[11]^ ASC is characterized by high aggressiveness, poor prognosis, and frequent advanced-stage diagnosis, limiting curative surgical options and necessitating chemotherapy dependence.^[12]^ Personalized cancer treatment prioritizes matching therapy intensity to mortality risk, yet no ASC-specific prognostic tools exist for chemotherapy patients. This study addressed this gap by developing and validating a CSS nomogram prediction model using SEER database data. By integrating 8 independent prognostic factors (age, household income, T/N stage, metastases, surgery, and primary tumor site), the model enables risk stratification (low/intermediate/high-risk subgroups) and supports tailored treatment decisions. This tool fills a critical unmet need in clinical prognostic assessment for ASC patients undergoing chemotherapy.

This study, based on the SEER database, meticulously screened and included data from a total of 3526 patients diagnosed with ASC. Among them, 40.53% of the patients received chemotherapy. After excluding cases with incomplete information, we ultimately identified 704 patients, who were randomly divided into a training group (493 cases) and a validation group (211 cases). In the training group, we employed univariate and multivariate Cox regression analyses to thoroughly investigate the key factors influencing the prognosis of ASC patients undergoing chemotherapy. The study results revealed that age, household income status, T stage, N stage, bone metastases, brain metastases, the primary site of the tumor, and surgery were all independent predictors of CSS in this specific patient subgroup. Based on these findings, we developed the first nomogram prediction model for this patient subgroup, which can simply and intuitively predict the 1-, 3-, and 5-year survival rates of patients. Through validation using the C-index and calibration curves, we confirmed a good agreement between the nomogram’s predicted outcomes and the actual observed data. Meanwhile, the assessment results from the ROC curves and AUC values indicated that the nomogram possessed excellent discriminatory ability, which was superior to that of single predictors. Additionally, the DCA further validated the high clinical practical value of this nomogram. To further enhance the precision and effectiveness of clinical decision-making, we also utilized this nomogram to construct a mortality risk stratification system, successfully subdividing patients into 3 risk subgroups: high-risk, intermediate-risk, and low-risk groups. The risk stratification system, derived from the nomogram’s total points using X-tile, serves not only to verify the model’s robust predictive performance – as evidenced by the significantly worse CSS in high-risk patients – but also to provoke critical clinical consideration. This stratification is clinically grounded, as it is based on 8 CSS-related independent predictors, including but not limited to the established TNM stage, thereby providing a more comprehensive prognostic profile by integrating factors like age and tumor size. Importantly, the identification of a high-risk group, which exhibited poor survival despite receiving chemotherapy, raises a pivotal question for personalized medicine: Could these patients, who are predicted to derive minimal survival benefit from standard chemotherapy, be considered for alternative treatment strategies upfront? This could spare them the significant toxicity of a potentially ineffective intervention. Thus, the model may serve as a practical tool to guide decisions regarding the necessity and intensity of chemotherapy.

In multivariate Cox regression, age was identified as an independent prognostic factor for CSS in ASC patients undergoing chemotherapy (*P* = .004), with older age significantly associated with poorer survival. This aligns with Liang et al retrospective study (n = 4600),^[9]^ where age stratification also showed a strong age-prognosis correlation (*P* < .001). The age-related prognostic disadvantage in elderly patients stems from multiple factors: physiological decline, including reduced liver/kidney function (impaired drug metabolism/clearance) and bone marrow atrophy (increased chemotherapy toxicity)^[13–15]^; age-associated comorbidities (e.g., smoking-related cardiovascular/pulmonary diseases) that complicate treatment and exacerbate condition^[16]^; and higher risk of malnutrition and functional deterioration, limiting treatment tolerance. Conversely, younger patients generally have better organ reserve and fewer comorbidities, enabling optimal treatment adherence and longer survival. Household income <$50,000 was associated with significantly worse CSS in ASC patients (*P* < .001), consistent with prior studies. Wu et al reported similar findings in extensive-stage small cell lung cancer with metastasis (*P* < .001),^[17]^ while an ADC study also identified low income as an independent prognostic factor (*P* < .001).^[18]^ Wang et al further linked low income to poor surgical compliance (*P* < .001), noting that higher income enables access to aggressive treatment, thereby improving survival.^[19]^ These results underscore socioeconomic factors – particularly household income – as critical determinants of cancer outcomes, with financial constraints limiting follow-up care and higher income acting as a guarantee of optimal treatment. Patients with ASC originating in the main bronchus had significantly shorter survival than those with tumors in other locations (*P* < .001), consistent with prior research. A retrospective study of 43,803 NSCLC patients confirmed poorer prognoses for main bronchus tumors (*P* < .001),^[20]^ with primary site identified as a key survival determinant. Potential explanations include: technical challenges of sleeve resection required for main bronchus tumors^[21]^; higher regional lymph node metastasis rates^[22]^; and proximity to vital organs, reducing radical resection rates and increasing peripheral organ involvement. These factors collectively contribute to worse outcomes, highlighting the need to consider tumor primary site in ASC treatment planning and prognosis assessment.

Multivariate Cox analysis identified brain and bone metastases as independent poor prognostic factors in chemotherapy-treated ASC patients, while liver (*P* = .985) and lung (*P* = .738) metastases showed no significant association. This differs from Xing et al finding of liver metastasis as a prognostic factor,^[23]^ possibly because chemotherapy is more effective against liver/lung metastases, diminishing their statistical power as independent predictors.^[24]^ Conversely, brain/bone metastases respond poorly to chemotherapy (e.g., limited blood–brain barrier penetration), leading to worse outcomes.^[25]^ These findings highlight the prognostic weight of specific metastatic sites in chemotherapy contexts. Future studies could integrate ctDNA and radiomics to explore underlying molecular mechanisms. Surgery is a critical treatment for ASC, particularly in early-stage patients. Among chemotherapy-treated ASC patients, those undergoing surgery had significantly better prognoses than nonsurgical patients (*P* < .001), consistent with Wu et al findings (HR = 0.45, *P* < .001).^[26]^ Surgical approach also impacts outcomes: a retrospective study of 244 ASC patients showed lobectomy achieved higher 5-year survival (35.0% vs 16.4%) and disease-free survival (29.5% vs 14.8%) compared to sublobar resection, with stage I patients deriving the greatest benefit.^[27]^

Radiotherapy is also one of the treatment modalities for ASC. The study by Ni et al^[28]^ found that adjuvant radiotherapy provided significant survival benefits for pulmonary ASC patients with pathological stages T3 and T4. This finding was further corroborated by Xing et al,^[23]^ who observed that advanced ASC patients who did not receive radiotherapy generally had poorer prognosis. Our study, based on a large-sample SEER database analysis, reveals the complex prognostic value of radiotherapy in the cohort of ASC patients treated with chemotherapy. A finding worthy of in-depth exploration is that while radiotherapy was significantly associated with poorer CSS in the univariate analysis, this association disappeared after multivariate adjustment for TNM stage (HR: 1.13, 95% CI: 0.87–1.47, *P* = .373). To clarify the reasons behind this apparent contradiction, we conducted subgroup analyses stratified by T and N stage, as well as interaction tests. The results uncovered significant stage-dependent heterogeneity in the treatment effect of radiotherapy (Figs. S1–S2, Table S1, Supplemental Digital Content, https://links.lww.com/MD/R323). The subgroup analyses showed that the effect of radiotherapy is not uniform but undergoes a fundamental reversal with the progression of the primary tumor (T) stage. In patients with T1 to T3 disease, radiotherapy consistently showed a trend towards increased risk of death (HR range: 1.48–2.20). However, in patients with T4 disease, this effect was reversed, showing a potential survival benefit (HR = 0.78, 95% CI: 0.48–1.28). This effect modification was confirmed by a formal statistical test, indicating a significant interaction between radiotherapy and T stage (*P* for interaction = .031). This finding perfectly explains the results of the multivariate analysis: when the statistical model attempted to estimate an “average” radiotherapy effect across all T stages, the clear detrimental effect in T1 to T3 patients and the potential beneficial effect in T4 patients counteracted each other, ultimately leading to the loss of significance for radiotherapy as a whole variable. This effect heterogeneity profoundly reflects a shift in the treatment intent of radiotherapy within the comprehensive management of ASC. For patients with T1 to T3 disease, where tumors are relatively localized, radiotherapy in this context was likely used as “adjuvant therapy” or “consolidation therapy” with curative intent. However, our data suggest that adding adjuvant radiotherapy to chemotherapy may not translate into a survival advantage and could even be detrimental, potentially due to overlapping treatment toxicities, potential negative impacts on the immune microenvironment, or patient selection bias. In stark contrast, for patients with T4 disease, who often have a high tumor burden and are accompanied by local symptoms, the application of radiotherapy leans more towards “palliative treatment.” At this advanced stage, the primary goal of radiotherapy is the effective management of disease-related symptoms such as refractory cough, hemoptysis, chest pain, and superior vena cava syndrome.^[29–31]^ Successful palliative treatment can significantly improve patients’ quality of life and performance status, which may enable them to tolerate systemic therapy for a longer duration, thereby indirectly translating into the observed trend towards survival improvement. Consequently, the potential survival benefit in T4 patients is more likely a manifestation of the excellent palliative efficacy of radiotherapy, rather than its direct curative effect on the tumor. The findings of this study have important potential clinical implications. For patients with pulmonary adenosquamous carcinoma receiving chemotherapy, radiotherapy should not be simplistically deemed “beneficial” or “harmful.” Instead, more refined decision-making is necessary. For early-stage (T1–T3) patients, the necessity of adding adjuvant radiotherapy to chemotherapy should be carefully evaluated. For advanced-stage (T4) patients, especially those with local symptoms, palliative radiotherapy remains a valuable treatment option worth considering. Future prospective studies are warranted to further validate this effect heterogeneity and to explore the underlying biological mechanisms.

In terms of tumor staging, increasing T or N stage – indicating tumor progression – was associated with progressively shorter survival duration, consistent with clinical experience.^[32–34]^ Since the Union for International Cancer Control first introduced the TNM staging system in 1968, it has undergone multiple revisions, with the International Association for the Study of Lung Cancer recently publishing the latest TNM staging criteria for lung cancer.^[35]^ The TNM system remains an indispensable tool for guiding clinical diagnosis, treatment, and prognostic assessment in lung cancer, underscoring its high accuracy in predicting outcomes. However, the TNM system primarily focuses on tumor characteristics and lacks integration of patient-related clinical factors. To address this, our study constructed a novel prognostic nomogram by incorporating T stage, N stage, and other independent prognostic factors. ROC curve analysis with AUC values demonstrated that this nomogram outperformed single-factor models (T or N stage alone) in predicting 1-, 3-, and 5-year survival, strongly confirming its superior prognostic accuracy. In the field of ASC research, previous evidence has suggested that male gender is associated with a poorer prognosis.^[36]^ These observations may be attributed to the smoking habits of males, particularly the cumulative effects of the quantity and duration of smoking, as smoking has been established as a significant risk factor for lung cancer.^[37]^ However, in our study, which focused on ASC patients receiving chemotherapy, we found no significant association between gender and prognosis (*P* = .087). This implies that, in the context of chemotherapy, patients’ responses to treatment are similar regardless of gender, and gender differences are not sufficient to influence treatment efficacy and prognosis.^[38,39]^

Gene mutations are also important factors affecting the prognosis of ASC patients. epidermal growth factor receptor (EGFR) mutations are particularly significant and often occur in the main components of the 2 histological types of ASC. Jia et al^[40]^ reported EGFR mutation frequency in adenosquamous lung carcinoma at 30% to 40%, and Wang et al^[41]^ found a higher detection rate (54.8%). EGFR-tyrosine kinase inhibitor therapy significantly improved prognosis in ASC patients with EGFR mutations. Song et al^[42]^ reported that in cases with EGFR mutations, the median progression-free survival was prolonged by 4.4 months after the use of EGFR-tyrosine kinase inhibitor. Lin et al^[43]^ similarly corroborated this observation. In addition to EGFR mutations, other genetic alterations such as K-Ras mutations, PI3K activation, or ALK gene rearrangements may also occur in ASC. However, due to limited gene mutation data in SEER and the rarity of ASC cases, their prognostic impact requires validation through larger, multicenter studies. PD-L1 positivity is relatively high in ASC patients: Liu et al^[44]^ reported 48.6% positivity at a 5% expression cutoff, while Wei et al^[45]^ observed a higher rate (64.7%). Efficacy evaluations of immune checkpoint inhibitors (ICIs) in 38 ASC patients showed objective response rate 23.7%, disease control rate 86.8%, median progression-free survival 5.47 months, and overall survival 24.10 months.^[45]^ Li et al,^[46]^ using real-world data from 46 patients across 11 centers, found ICIs-based treatment yielded objective response rate 28%, median progression-free survival 6.0 months, and overall survival 24.7 months. These findings suggest ICIs significantly improve prognosis in PD-L1-positive ASC. However, comparative studies on ICIs efficacy differences are limited, and current evidence is insufficient to determine the optimal ICIs. Larger clinical studies are needed to guide clinical selection.

Certainly, this study has several limitations. First, as a retrospective analysis based on the SEER database, it is inherently prone to potential selection bias. Second, despite the relatively large number of events, the overall sample size remains constrained by the rarity of ASC, which may affect the generalizability of our findings. Third, and most critically, the SEER database lacks crucial molecular profiling data, such as EGFR mutations and PD-L1 expression status. Consequently, our nomogram, derived from a chemotherapy-treated cohort, reflects the “chemotherapy era” and may have limited applicability and require recalibration for patients receiving modern targeted therapies or immunotherapy. Nonetheless, this model may still hold reference value for analyzing historical data, for patients lacking molecular test results, or in resource-limited settings. Fourth, detailed information on specific chemotherapy regimens, imaging findings, and smoking history is unavailable, which could serve as unmeasured confounders. Finally, the performance and reliability of this nomogram necessitate external validation in independent cohorts or prospective studies. Despite these limitations, our study conducted in-depth exploration within a relatively large-scale cohort, ensuring the richness of data and comprehensiveness of patient information, which provided robust support for the study’s reliability and depth. The core contributions of this study lie in our innovative development of a prognostic prediction model for ASC patients undergoing chemotherapy and the subsequent creation of a nomogram based on this model. A key future direction is to integrate clinicopathological features with molecular profiling to build a more robust prognostic tool suited for the era of precision medicine.

## 5. Conclusion

This study relied on the SEER database to screen relevant data on ASC and employed univariate and multivariate Cox regression analysis methods to thoroughly investigate the risk factors influencing the prognosis of ASC patients undergoing chemotherapy. Through in-depth analysis, we identified 8 key variables: age, household income status, T stage, N stage, bone metastasis status, brain metastasis status, surgery, and primary tumor site. Building upon these findings, we innovatively utilized these independent predictors to construct the first nomogram model specifically designed for evaluating the prognosis of ASC patients undergoing chemotherapy. To ensure the accuracy and practicality of the model, we rigorously validated it in both the training group and the testing group. The validation results demonstrated that the model exhibits a high degree of predictive reliability, providing clinicians with a valuable reference tool for predicting the prognosis of ASC patients undergoing chemotherapy. Finally, through the risk stratification system developed using the nomogram, we effectively divided ASC patients undergoing chemotherapy into 3 subgroups. Kaplan–Meier analysis revealed significant differences among these subgroups, indicating the model’s excellent predictive capability and enabling better management of cancer patients.

## Author contributions

**Validation:** Jiarun Fan, Maoping Wang.

**Writing – original draft:** Jinyuan Xiao.

**Writing – review & editing:** Xiaoliang Yuan.

## Supplementary Material


