# Supplementary material for: Prognostic nomogram for cancer-specific survival in adenosquamous carcinoma of lung patients treated with chemotherapy: A SEER-based retrospective cohort study
Source: Medicine (Baltimore). 2026 Feb 13;105(7):e47538. doi: 10.1097/MD.0000000000047538 (PMC12908825; doi:10.1097/MD.0000000000047538)
Supplement: Supplementary file 1 [file medi-105-e47538-s001.pdf]

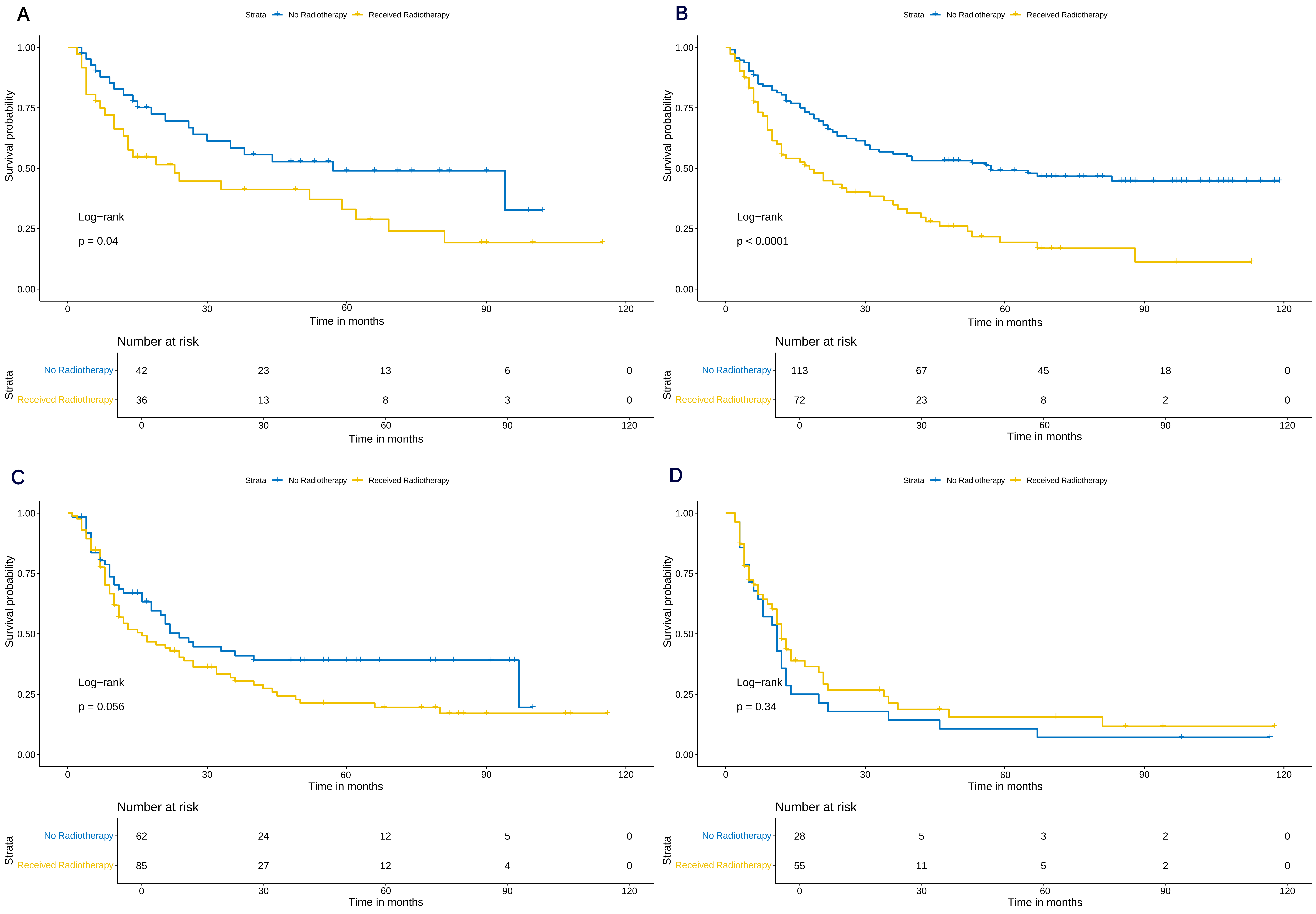
A
Strata No Radiotherapy Received Radiotherapy
Survival probability
Log−rank
p = 0.04
Time in months
Number at risk
No Radiotherapy 42 23 13 6 0
Received Radiotherapy 36 13 8 3 0
B
Log−rank
p < 0.0001
No Radiotherapy 113 67 45 18 0
Received Radiotherapy 72 23 8 2 0
C
Log−rank
p = 0.056
No Radiotherapy 62 24 12 5 0
Received Radiotherapy 85 27 12 4 0
D
Log−rank
p = 0.34
No Radiotherapy 28 5 3 2 0
Received Radiotherapy 55 11 5 2 0

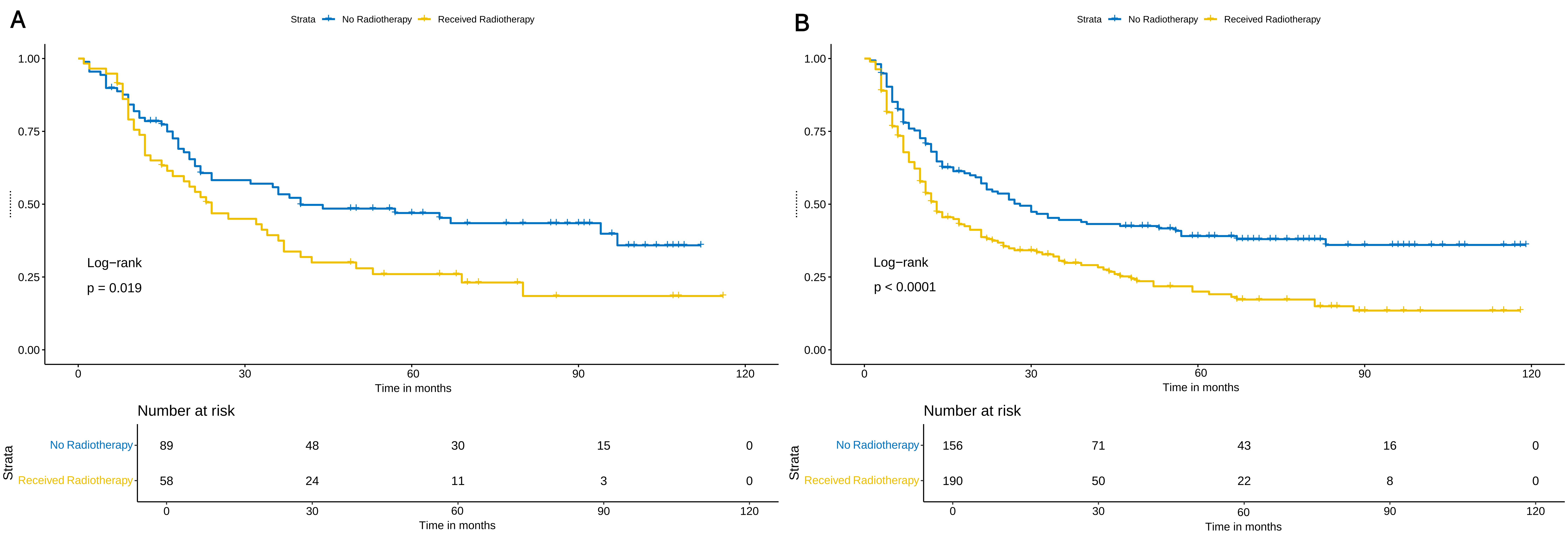

A
Strata
No Radiotherapy
Received Radiotherapy
1.00
0.75
0.50
0.25
0.00
Log−rank
p = 0.019
0
30
60
90
120
Time in months
Number at risk
Strata
No Radiotherapy
Received Radiotherapy
89 48 30 15 0
58 24 11 3 0
Time in months
B
Strata
No Radiotherapy
Received Radiotherapy
Log−rank
p < 0.0001
Time in months
Number at risk
Strata
No Radiotherapy
Received Radiotherapy
156 71 43 16 0
190 50 22 8 0
Time in months

Supplementary table 1 Subgroup Analysis of the Association Between Radiotherapy and Cancer-Specific Survival

**Subgroup**	**Patients, n**	**CSS events, n**	**HR (95% CI)**	***P* value**	***P* for interaction**
**T-stage**					0.031
**T1**	78	45	1.84 (1.02-3.32)	0.042	
**T2**	185	113	2.20 (1.51-3.19)	<0.001	
**T3**	147	100	1.48 (0.99-2.23)	0.059	
**T4**	83	67	0.78 (0.48-1.28)	0.330	
**N-stage**					0.156
**N0**	147	92	1.63 (1.08-2.46)	0.020	
**N1-3**	346	233	1.73 (1.33-2.25)	<0.001	
